# Outpacing the pneumococcus: Antibody dynamics in the first few days following pneumococcal capsular antigen stimulation

**DOI:** 10.1038/s41598-018-33735-x

**Published:** 2018-10-18

**Authors:** Sheila Z. Kimaro Mlacha, Anne Warira, Hellen Gatakaa, David Goldblatt, J. Anthony G. Scott

**Affiliations:** 1 Kenya Medical Research Institute – Wellcome Trust Research Programme, Kilifi, Kenya; 2 Respiratory & Meningeal Pathogens Research Unit, University of the Witwatersrand, Johannesburg, South Africa; 3 Great Ormond Street Institute of Child Health, University College London, London, UK; 4 London School of Hygiene & Tropical Medicine, London, UK

## Abstract

Children in developing countries are frequently exposed to the pneumococcus, but few develop invasive pneumococcal disease (IPD). We test the hypothesis that natural variation exists in the rapidity of IgG responses following exposure to pneumococcal polysaccharides, and that these differences are sufficiently great to affect susceptibility to and outcome of IPD. We recruited children aged 24–36 months, who had recovered from IPD, and age-matched healthy controls and vaccinated them with 1 dose of the 23-valent PPV to mimic natural exposure. We collected serum samples after vaccination and analysed the dynamics of anti-polysaccharide antibody responses to several capsular antigens. Mean IgG response times to different serotypes were 6.4–7.3 days, with standard deviations of 0.9–1.85 days, suggesting a natural range in response times of up to 7 days. Serotype 1 elicited the largest fold-rise, serotype 23F the smallest. The proportion of responses achieved by day 7 was similar in children with a history of IPD and healthy children. There was considerable natural variation in the rapidity of anti-capsular IgG responses extending over 4–7 days. There was no evidence to suggest that children who have experienced IPD respond more slowly to heterologous pneumococcal capsular antigens than do healthy children.

## Introduction

It has been proposed that neutralizing serum IgG antibodies are sufficient to protect against many diseases including those caused by *Streptococcus pneumoniae* (pneumococcus)^1^. Pneumococci are killed when serum containing high levels of antibodies attach to the polysaccharide, attracting and activating serum complement proteins to form C3 and C5 complexes. The antibody-complement complexes bind receptors on phagocytic cells activating them and leading them to engulf and digest the pneumococcus (opsonophagocytosis). The protective role of these circulating antibodies in pneumococcal infections has been demonstrated in studies involving both passive^2–6^ and active immunization^7–13^. The minimum protective serum concentrations of pneumococcal anti-capsular antibodies has been estimated using aggregate correlates of protection to be 0.20 µg/ml^14^ or 0.35 µg/ml^15^ depending on the ELISA method used. Recently, serotype-specific correlates of protection of 0.78–2.83 µg/ml for serotypes 1,3, 7F, 19A, 19F and 0.14–0.20 µg/ml for serotypes 6A, 6B, 18C and 23F have been defined^16^.

In contrast to the notion of an absolute level of serum IgG required for protection, other studies have shown that some individuals with ‘non-protective’ or modest baseline antibody levels were still protected against invasive pneumococcal infections^17,18^. Moreover, in a randomized immunogenicity study of American Indian children given a pneumococcal conjugate vaccine (PCV7), a child developed invasive pneumococcal disease despite adequate levels of IgG antibody (4.98 μg/ml) against the disease-causing serotype^19^. These results suggest that the absolute amount of serum antibody is not the only immunological yardstick of a protected state. Immunological memory has been suggested as a mechanism to explain sustained protection in the absence of detectable antibody^17,20–23^. However, given the accumulation of vaccine failures after memory-inducing vaccines, such as PCV, meningococcal serogroup C conjugate vaccine and *Haemophilus influenzae* type b conjugate vaccine^19,24–28^, the presence of immune memory does not guarantee protection either.

A plausible explanation for disease occurring in primed individuals with existing antibody levels is that the booster response was not sufficiently rapid to prevent the invasion and bacterial multiplication that usually occurs within a few days of colonization^29^. In the race between the expansion of pneumococci in the body and the immune response of the host, a delay of 24–48 hours in the activation of B cells and their differentiation into antibody-producing cells may be clinically significant. It is therefore possible that the dynamics of a limited anti-capsular response, either in a primed or unprimed individual, might be an important factor discriminating between those who achieve control of infection and those who do not.

We carried out two studies to investigate the following related questions: (1) What is the natural variation in the speed of human IgG response following exposure to pneumococcal capsular antigens? (2) Do individuals who are susceptible to IPD respond less rapidly with IgG following antigen stimulation than normal children? For this second study we took children who had previously had IPD to represent those with demonstrated ‘susceptibility’ although we excluded an analysis of their response to the previously infecting serotype as this could confound our analysis either due to recent memory resulting in a rapid response or due to hyporesponsiveness to the homologous serotype blunting/slowing the response^26^. The 23-valent Pneumococcal Polysaccharide Vaccine (PPV23) was used as the immunogen in both studies to mimic the natural infection process. We evaluated responses to 5 common serotypes found in Kilifi district: 1, 6B, 14, 19F and 23F. Serotypes 6B, 14, 19F and 23F were among the top 6 most frequently carried serotypes in children aged <5 years in Kilifi district^30^. The incidence of serotype 1 disease was high at 26% in children aged 24–59 months in Kilifi district^31^.

## Results

### Study 1

#### Geometric mean antibody concentrations

**Figure 1:**
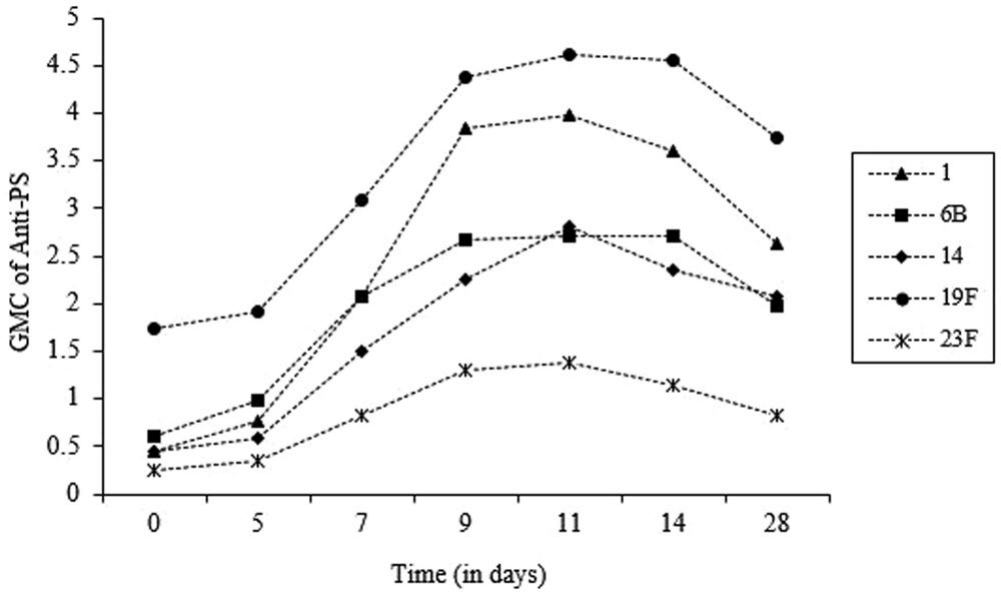
Geometric mean concentrations of antibodies to pneumococcal serotypes 1, 6B, 14, 19F and 23F, before and after immunization with polysaccharide vaccine in healthy children (*n* = 36).

Forty children were recruited (median age 29 months; range 24–35), of whom 18 (45%) were male. Four children withdrew from the study before completing it. Before vaccination, the GMCs varied by serotype from 0.17 μg/ml, for serotype 23F, to 1.54 μg/ml, for serotype 19F. Serotype 1 elicited the greatest mean increase (9.2-fold) by day 11, serotype 19F the smallest (2.7-fold). After day 11, the anti-capsular antibody concentrations started to decline, but remained above the pre-vaccination concentration by day 28 (Fig. 1). A negative correlation (r = −0.35 to −0.61, *p* = 0.0009–0.036) between antibody fold increase and pre-vaccination antibody titre was found for all serotypes.

#### Dynamics of IgG antibody production

**Figure 2:**
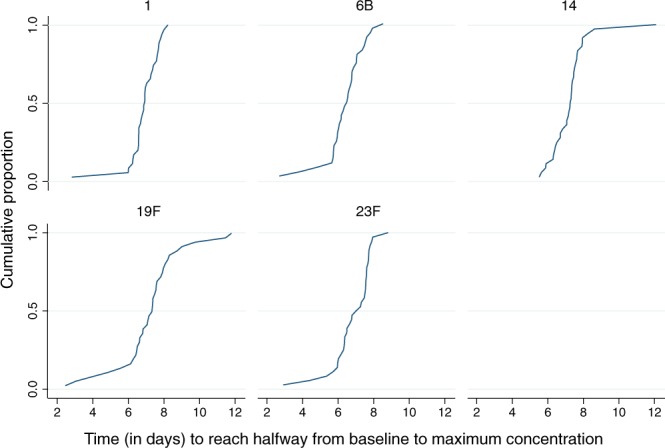
Cumulative distribution curves representing the proportion of children reaching the response threshold (half of the maximum) against time since vaccination for serotype 1, 6B, 14, 19F and 23F.

We then measured whether there was variation in the rapidity of immune responses. Response times (defined as time to reach half of the difference between baseline and maximum concentration) were normally distributed and the means ranged from 6.4, for serotype 6B, to 7.3 days, for serotype 14 (Table 1). Response times varied by serotype (*p* = 0.007) and subject (*p* < 0.0005). Standard deviations were in the range 0.91–1.85 for each serotype suggesting a response time range of approximately 4–7 days. The widest dispersion of response times by subject was seen for serotype 19F (SD 1.85). The cumulative proportion of children that had reached the response threshold (half the maximum) is shown in Fig. 2. By day 5, 31% had reached this threshold for serotype 6B but for all other serotypes less then 20% of children had reached the threshold. By day 6, the proportion that had reached this threshold was 60%, 72%, 33%, 39% and 50% for serotypes 1, 6B, 14, 19F and 23F, respectively. By the 8^th^ day all had reached the threshold for all serotypes except 19F for which the proportion was 78%; the remaining children reached the threshold by day 11. Therefore, most children mounted IgG responses 6 to 7 days after vaccination but there was considerable variation over a period of days as shown by the lower and upper tails on the curves representing fast and slow responders, respectively. We found that the gradient of the cumulative response curve was steepest around day 7, and that for most children, antibody concentrations reached peak levels 11 days after exposure to polysaccharide. Days 7 and 11 were therefore selected as the optimum sampling days for Study 2.

**Table 1:** Distribution of response times and increases in geometric mean concentrations of IgG antibodies to 5 polysaccharides of Streptococcus pneumoniae from pre-vaccination to day 11 in young children (*n* = 36).

Serotype	Distribution of response times (days)			Fold increase
	mean	Sd	cv*	(day11/day0)
1	6.89	0.91	0.13	10
6B	6.41	1.09	0.17	4.1
14	7.25	1.1	0.15	6.2
19F	7.21	1.85	0.26	2.8
23F	6.83	1.11	0.16	6.5

*cv, coefficient of variation.

### Study 2

Sixty children were recruited, of whom 30 had a history of IPD. In the Prior IPD group and the Healthy Control group the median ages were 37 and 36 months, respectively, and there were 21 (70%) and 11 males (36%), respectively. Two children in the Healthy Control group and three in the Prior IPD groups withdrew from the study. Among the Prior IPD group, we excluded eight children from the analysis of serotype 1, three from serotype 6B and 2 from 14, 19F and 23F because they had previously had IPD of these serotypes.

#### Geometric mean antibody concentrations

The distribution of pre-vaccine serotype-specific IgG concentrations for the Prior IPD group and Healthy Controls is shown in Table 2. There were no significant differences in baseline GMCs between the Prior IPD group and the Healthy Control Group (P > 0.5) for all serotypes (Table 2). The proportion of children with anti-capsular antibody ≥0.35 µg/ml was also similar; 37% vs. 42% (serotype 1), 54% vs. 61% (serotype 6B), 36% vs. 36% (serotype 14), 68% vs. 82% (serotype 19F) and 16% vs. 18% (23F) in Prior IPD and Healthy Control groups, respectively (Supplementary Figure 1). The serotype with the highest pre-vaccination antibody GMC was 19F in both groups.

**Table 2:** Geometric mean concentrations (µg/ml) of antibodies to pneumococcal serotypes 1, 6B, 14, 19F and 23F before and after immunization with polysaccharide vaccine in children with (Prior IPD group) and without (Healthy Control group) a history of IPD.

Serotype	Group	Geometric mean antibody level [mcg/ml (95% CI)]			
		Pre-vaccination	Post-vaccination	Fold increase	P
		day0	day11	(day 11/day 0)	value^b^
1	Prior IPD (*n* = 19)	0.25(0.17–0.37)	2.70**(1.72–4.23)	10.85	
	Healthy Control (*n* = 28)	0.30(0.20–0.44)	4.77**(3.60–6.72)	16.06	0.005
6B	Prior IPD (*n* = 24)	0.34(0.22–0.52)	1.05*(0.6–1.84)	3.1	
	Healthy Control (*n* = 28)	0.49(0.33–0.74)	1.69*(1.15–2.49)	3.45	NS
14	Prior IPD (*n* = 25)	0.20(0.12–0.32)	1.01*(0.54–1.86)	5.05	
	Healthy Control (*n* = 28)	0.17(0.11–0.26)	1.08*(0.56–2.09)	6.35	NS
19F	Prior IPD (*n* = 25)	0.68(0.40–1.15)	2.37*(1.47–3.82)	3.49	
	Healthy Control (*n* = 28)	0.60(0.38–0.95)	1.75*(1.16–2.66)	2.92	NS
23F	Prior IPD (*n* = 25)	0.10(0.06–0.16)	0.87**(0.55–1.36)	8.7	
	Healthy Control (*n* = 28)	0.09(0.06–0.13)	0.65**(0.42–1.01)	7.2	NS

IPD = Invasive Pneumococcal Disease; *P < 0.002; **P < 0.0006 (Wilcoxon’s non-parametric test); ^b^*P* < 0.05; ns = not statistically significant (*t*-test) for the comparison of fold increase between Prior IPD and Healthy Control groups.

There was a significant increase in mean antibody concentrations from baseline to day 11 in both groups for all serotypes (*P* = 0.005) (Table 2), with serotype 1 eliciting the greatest mean increase (>10-fold rise). The mean increases were similar between the two groups except for serotype 1, for which the Healthy control group had a significantly higher increase than the Prior IPD group (*P* = 0.005) after 11 days. The proportion of children with anti-capsular antibody ≥0.35 µg/ml by day 11 was >50% for all groups and serotypes (Supplementary Figure 1).

#### Association of dynamics of IgG antibody production and susceptibility to IPD

**Figure 3:**
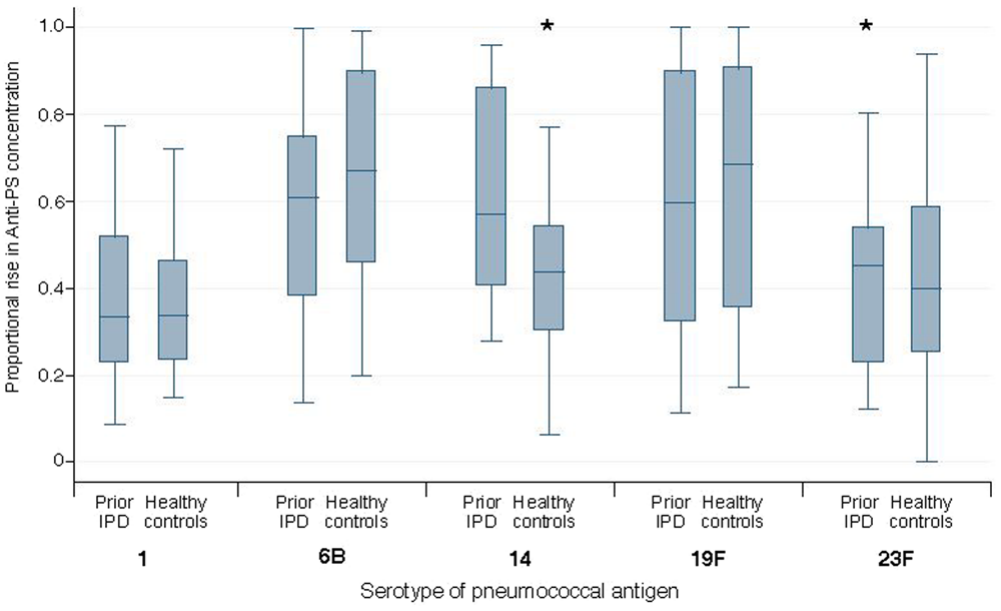
Proportion of the total antibody concentration rise achieved by 7 days for five capsular antibodies among Prior IPD and Healthy Control groups. Box plots show 25^th^ and 75^th^ percentiles, error bars show 10^th^ and 90^th^ percentailes and median values are shown as (−). Proportional rise = C_7_ − C_0_*/*C_11_ − C_0_ where C_0_, C_7_ and C_11_ represent concentrations on day 0, 7 and 11 respectively. IPD = Invasive Pneumococcal Disease *P < 0.05 (Wilcoxon rank sum test).

The mean proportion of responses achieved by day 7 was significantly higher in the Prior IPD group than the Healthy Control group for 2 of the 5 serotypes (14 and 23F, Fig. 3).

## Discussion

The purpose of the study was to investigate the dynamics of the humoral response to a representative set of *Streptococcus pneumoniae* capsular polysaccharides in order to determine if there is natural variation in the speed of antibody production in the days following exposure to pneumococcal polysaccharide antigens and if this variation is associated with prior IPD, as an indicator of susceptibility to bacterial invasion. If the hypothesis were true, it would identify an immunological marker that could be used to screen for patients at particularly high risk of IPD.

This idea has been explored in a previous study in mice, where the authors found a significant difference in the dynamics of antibody response to a *Cryptococcus neoformans* protein between mice that survived the infection and those that died^32^. Consistent with the sub-acute nature of cryptococcal infection, they found that non-survivors mounted strong humoral responses during the acute phase, whereas survivors produced antibodies later, during the chronic phase of the infection.

In human subjects, a study by Sorensen *et al*.^33^ found no differences in geometric mean antibody concentration to polysaccharide antigens between healthy adults and infection-prone adult patients when immunized with PPV23. We hypothesized that, in a comparison of the speed of antibody response after vaccine exposure to a polysaccharide antigen, children with a history of invasive pneumococcal infection would have inherently slower anti-capsular response times than healthy children and that this inherently slow response may have been a contributory factor in the development of their episode of disease.

We found that even though individuals responded with widely different dynamics following stimulation with pneumococcal polysaccharide antigens, the group of children with a history of IPD did not respond more slowly than the control group. In fact, for two out of the five serotypes, the IPD group responded more quickly than the healthy controls.

This unexpected result may have come about for a number of reasons. Firstly, the study does not include children who suffered with fatal IPD and it may be that slow responses are associated with outcome of IPD rather than with the probability of developing disease. To study IPD outcome would require a prospective cohort study where the exposure (antibody response times) was established at baseline for a population of such size as to yield a useful number of cases of IPD. Even in Kenya, IPD is an uncommon event and such a cohort would be impracticable.

Secondly, the hypothesis may be true, but only for the homologous serotype. We did not study homologous serotypes because: 1) the prior episode of IPD may have primed for a memory response giving prior IPD cases an apparent advantage over healthy controls for that serotype and 2) the prior episode of IPD may result in hyporesponsiveness to the homologous serotype, as has been shown previously^26^. We examined the broader hypothesis that children with IPD are likely to have slow responses to polysaccharide antigens in general. Again, a cohort study is the only design that could overcome this limitation, if it were practicable.

Thirdly, the association may be confounded. The vigorous antibody responses observed have clearly been shaped by prior natural exposure to pneumococcal antigens in nasopharyngeal carriage. Children who are more frequently exposed to pneumococci may be more likely to develop IPD but they are also more likely to have acquired a wide repertoire of memory responses to different serotypes.

Our results do show that most 2–3 year olds have evidence of prior exposure to these antigens and yet the study was conducted in a period before the introduction of routine immunisation with PCV (PCV10 was introduced in January 2011) and PPV23 was not in use in children at any stage. All the children studied were at least 2 years old and were living in an area with high pneumococcal carriage^30^. The antibody responses of these vaccine-naïve children to a first dose of PPV23 were rapid, and of IgG isotype, characteristic of memory antibody responses. Pre-vaccination antibody titres were high in children from both study groups and relatively high proportions of children had already reached the putative protective threshold of ≥0.35 µg/ml, which was derived for vaccine-induced antibody, on the basis of natural exposure alone; this was especially true for serotypes 19F and 6B (Supplementary Figure 1). These two serotypes were also among the top 2 most frequently carried serotypes in children aged <5 years in Kilifi district^30^. The development of serum antibodies in response to nasopharyngeal colonization has been described previously in a similar environment in Thailand^34^. Confounding by prior exposure could have been overcome by studying younger children but, unfortunately, children <2 years of age respond poorly to PPV23 since they lack the mature B lymphocytes necessary for a T-cell-antibody-mediated immunity^35^. Although this is a convincing explanation in our setting, studies of response dynamics following *Haemophilus influenzae* Type b vaccine were shown to be similar in children with and without significant prior HbPs antibody levels^36^.

Fourthly, the differences in rapidity of response in the first few days after vaccination might have been better delineated by the characterization of IgM responses in antibody forming cells in peripheral blood, as has been used previously^37^.

Finally, it may be argued that the stimulus used, PPV23, is not a sufficient mimic of natural exposure. We considered the use of PCV in this study but the observations that post-primary immune responses in young infants are considerably higher than naturally induced immune responses in older children, even in settings with high carriage, suggest that PCV is an ‘unnatural’ stimulus^38^.

Despite these limitations and their potential impact on the measured association between response rapidity and prior IPD in *Study 2*, the results of *Study 1* do present a very plausible case for the potential role of response times in determining clinical outcomes. Following stimulus with most serotypes, adequate responses appear in a small fraction of children within 5 days and are evident in most children by the completion of 9 days; for serotype 19F this window stretches from 3.5 to 11 days. Given the rapidity of clinical development of IPD^29^ and its rapid evolution to death – often within 24–48 hours – the variation in response times for the key mechanism of acquired immunity does suggest that host responses may be a significant determinant of outcome.

In conclusion, our results showed wide variation in the rapidity of response to pneumococcal polysaccharide antigens suggesting that there is a critical time window in which some individuals may be more prone to invasive disease than others. Although we did not find direct evidence that the response-time is associated with susceptibility to disease, such an association is methodologically challenging to demonstrate and the failure to do so within the present design provides little assurance that response-times can be ignored in the pathogenesis of IPD and the dynamics of the acquired immune response.

## Methods

### Study population

In *Study 1* a convenience sample of 40 children aged 24–36 months was selected from among healthy siblings of patients admitted to the paediatric ward in Kilifi District Hospital and who lived within 30 km of the hospital. Subjects were excluded if they had any of the following: (1) a history of invasive pneumococcal infection including pneumonia, bacteremia, or meningitis documented at the hospital; (2) a history of previous vaccination with any pneumococcal vaccine; (3) receipt of any other vaccine in the last 2 months; (4) admission to hospital in the last 3months; (5) malnutrition, as defined by a weight-for-age z-score of <−3.0; or (6) HIV infection. The study was completed before the introduction of Pneumococcal Conjugate Vaccine (PCV) into the routine immunisation system in Kenya.

In *Study 2*, thirty children who had recovered from an episode of invasive pneumococcal disease (the Prior IPD group) were compared with 30 healthy age-matched children who had not had IPD (the Healthy Control group). Controls were selected at random from a cohort study investigating environmental and genetic susceptibility to invasive pneumococcal disease in Kilifi District Hospital. An episode of IPD was defined as admission to hospital with cultures of blood, cerebrospinal fluid (CSF) or pleural aspirate that grew *S. pneumoniae*. The exclusion criteria employed in study 1 were also used in study 2 except that for the Prior IPD group, history of invasive pneumococcal disease was an inclusion criterion.

The protocol was approved by the KEMRI/National Ethical Review Committee and written informed consent for the children’s participation was obtained from their parents or guardians. Recruitment of the study participants took place between October 2004 and December 2004 for study 1 and November 2005 and June 2006 for study 2. Serum sampling took place soon after patient recruitement and all serum collection was completed within the same years of recruitment. All experiments were completed within two years of sample collection and were performed in accordance with relevant guidelines and regulations. This study was registered on the www.clinicaltrials.gov website on 8^th^ March 2018 (Identifier: NCT03460730).

### Immunization and serum sampling

All children in both studies received a single sub-cutaneous dose (0.5 ml) of PPV23 (Merck, Sharp and Dohme, West Point, PA). In Study 1, blood samples (2 ml) were drawn from each child immediately before vaccination, then at 5, 7, 9, 11, 14 and 28 days after vaccination. Analysis of this first study suggested that the optimal days for observing the speed of response, if constrained to two sample points, were days 7 and 11. In Study 2 therefore, blood samples were drawn from each child before vaccination and 7 and 11 days after the vaccination. Serum was separated from the blood samples and stored at −80 °C until tested. Pre- and post-immunization sera from each individual were tested on the same ELISA plate.

### Pneumococcal antibody enzyme-linked immunosorbent assay (ELISA)

Concentrations of IgG antibodies to pneumococcal serotypes 1, 6B, 14, 19F and 23F were measured using the 22F inhibition ELISA method as previously described^39,40^. In brief, sera from each subject were pre-absorbed with cell wall polysaccharide (10 μg/ml; Statens Serum Institut, Copenhagen, Denmark) and 22F, to remove antibodies reactive with non-type-specific cell wall components of *S. pneumoniae* and other contaminants. The samples were added to a series of wells coated with the appropriate type-specific pneumococcal antigen (purchased from American Type Culture Collection). Antibodies specific for the capsular polysaccharides were detected using alkaline phosphatase-conjugated goat anti-human IgG antibody and the results were calculated on the basis of the officially assigned IgG values of the 89SF reference serum^41^. A quality control serum sample (pooled high-titred sera from adults immunized with PPV23) was included on each plate. As an additional control, contamination of PPV23 by the pneumococcal protein antigen, Pneumococcal Surface Antigen A (PsaA), was examined using a standardized ELISA for anti-PsaA IgG^42,43^. The ELISA assays were performed at the KEMRI-Wellcome Trust Research Programme in Kilifi, Kenya.

### Analytical methods

ELISA results were analyzed by a four-parameter logistic-log curve fit program (ELISA v.2.15; Center for Disease Control and Prevention, Atlanta, USA.) and expressed in μg/ml. Antibody concentrations were expressed as geometric mean concentrations (GMC) with 95% confidence intervals (CI). Comparisons of GMCs at each time point against day 0 were made using the Wilcoxon signed-rank test. Between-group comparisons (Prior IPD vs. Healthy Control groups) of GMCs were made using the Mann-Whitney U test as the distribution of the two populations was not log-normal and also due to the small sample size. The fold-change values were compared using a student’s *t*-test. All statistical analyses were performed using STATA 14.0 (StataCorp, College Station, TX).

The dynamics of the antibody response were analyzed in a similar manner in the two studies. In Study 1, individual response curves for each subject and each antigen were drawn and the pre-vaccination and the maximum concentrations were then defined. The outcome variable “response threshold” was defined as the time in days to make at least one half of the total IgG response. The total IgG response is the difference between the maximum observed antibody concentration and the starting concentration. Differences in mean response times were tested by ANOVA. In Study 2, the response was defined from individual response curves, as the rise in antibody concentration between the starting concentration (at day 0) and maximum concentrations (at day 11). The proportion of this response that had taken place by day 7 (C_7_) was estimated as follows: $proportionalrise={\rm{C}}7-{\rm{C}}0/{\rm{C}}11-{\rm{C}}0$ where C_0_, C_7_ and C_11_ represent concentrations on day 0, 7 and 11 respectively. For the Prior IPD group, we did not evaluate the responses of children to the (homologous) serotype that had caused their episode of disease, since this would represent an anamnestic response or demonstrate hyposresponsiveness hence confounding the results.

## Electronic supplementary material

Dataset 1
