# Supplementary material for: Outpacing the pneumococcus: Antibody dynamics in the first few days following pneumococcal capsular antigen stimulation
Source: Sci Rep. 2018 Oct 18;8:15376. doi: 10.1038/s41598-018-33735-x (PMC6193966; doi:10.1038/s41598-018-33735-x)

**Outpacing the pneumococcus: Antibody dynamics in the first few days following pneumococcal capsular antigen stimulation**

**Running title:** Dynamics of IgG antibody production

###### Authors

Sheila Z. Kimaro Mlacha^1,2*^, Anne Warira^1^, Hellen Gatakaa^1^, David Goldblatt^3^, J. Anthony G Scott^1,4^

# Author Affiliations

^1^Kenya Medical Research Institute – Wellcome Trust Research Programme, Kilifi, Kenya

^2^Respiratory & Meningeal Pathogens Research Unit, University of the Witwatersrand, Johannesburg, South Africa

^3^Great Ormond Street Institute of Child Health, University College London, London, UK

^4^London School of Hygiene & Tropical Medicine, London, UK

^*^ Corresponding author: Sheila Zena Mlacha Kimaro, Respiratory and Meningeal Pathogens Research Unit, Chris Hani Baragwanath Hospital, Johannesburg, 2013 South Africa, Tel: +27 714058664, Email: [shezekimla@gmail.com](mailto:shezekimla@gmail.com)

Proportion (%) of Prior IPD cases and Healthy Controls achieving the putative protective concentration (of ≥0.35µg/ml) by day and serotype


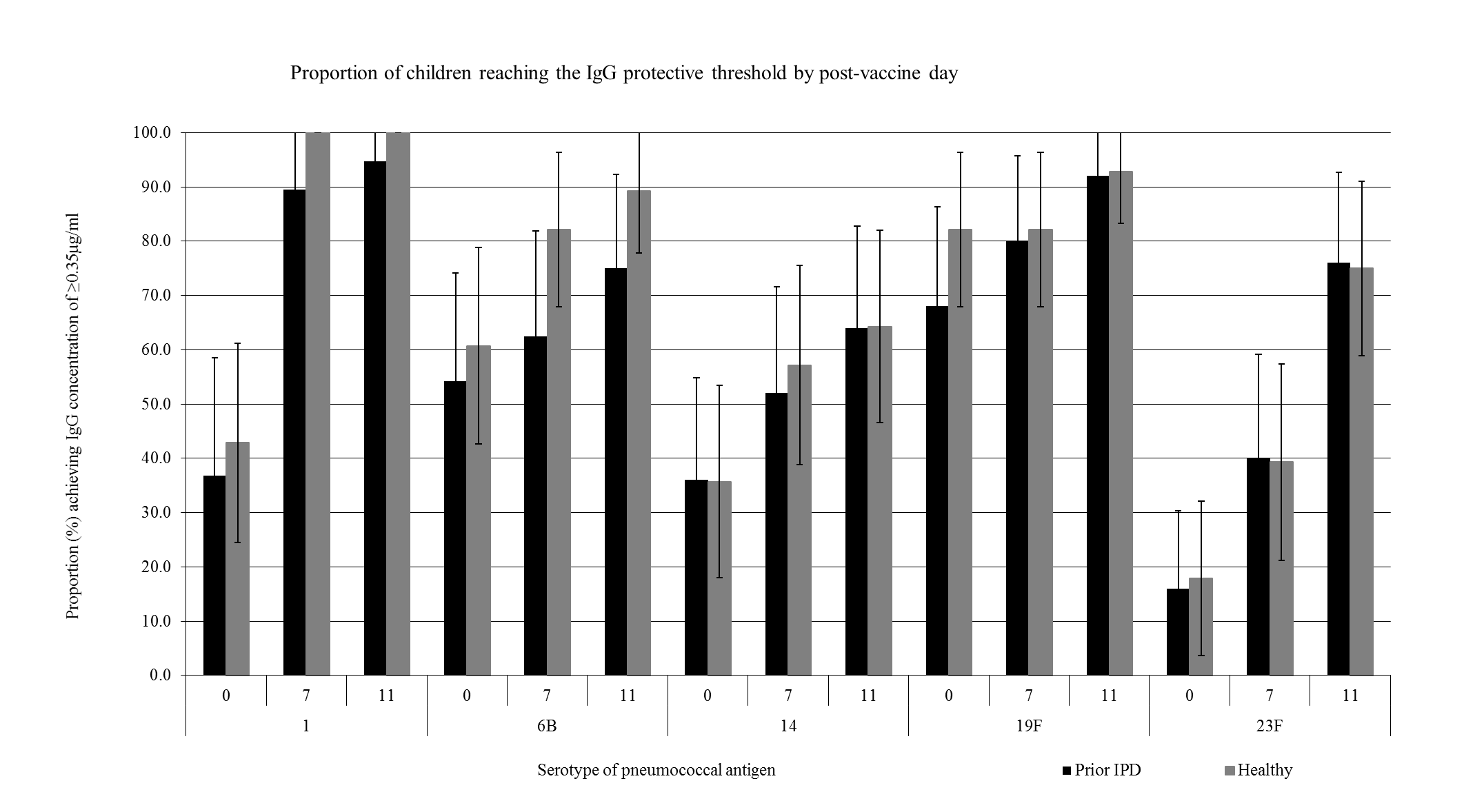

Supplement: Supplementary file 1 — Dataset 1 [file 41598_2018_33735_MOESM1_ESM.docx]
